# Supplementary material for: Rapid turnover of life-cycle-related genes in the brown algae
Source: Genome Biol. 2019 Feb 14;20:35. doi: 10.1186/s13059-019-1630-6 (PMC6374913; doi:10.1186/s13059-019-1630-6)
Supplement: Supplementary file 3 — Protein alignments, corresponding Gblocks html file and CDS sequences for the lineage conserved gametophyte-biased genes. (ZIP 2109 kb) [file 13059_2019_1630_MOESM3_ESM.zip › Con_GA_alignments/Ec-10_005660.aa_ali.fasta-gb.htm]

Ec-10\_005660.aa.fasta.trans.aa\_ali.fasta


## Gblocks 0.91b Results

Processed file: **Ec-10\_005660.aa.fasta.trans.aa\_ali.fasta**  
Number of sequences: **2**  
Alignment assumed to be: **Protein**  
New number of positions: **525** (selected positions are underlined in blue)

```
                         10        20        30        40        50        60
                 =========+=========+=========+=========+=========+=========+
Ec-10_005660     MASPAGTFSSCSTQRGASRRSWNGRERDVLHSRCRSLPPRRRVITRSRSRSTCRSAGGSS
MSTRG.40154.1    ------------------------------------------------------------
                                                                             


                         70        80        90       100       110       120
                 =========+=========+=========+=========+=========+=========+
Ec-10_005660     PFDGQSAASEELKPAQHNEKLTRSTGRSSRIRVGVRIRPPFVSEVDYQKRSGGYRPAVQI
MSTRG.40154.1    ------------------------------------------------------------
                                                                             


                        130       140       150       160       170       180
                 =========+=========+=========+=========+=========+=========+
Ec-10_005660     SRQGCVDLIASGRQRSFPFDYAFDATCQQHELYQSLAAPVVDGCLTGFNGLIMAYGQTAS
MSTRG.40154.1    ----------------------------QDDIYRSLAAPVVEGCLAGLNGLILAYGQTAS
                                             ################################


                        190       200       210       220       230       240
                 =========+=========+=========+=========+=========+=========+
Ec-10_005660     GKSHSMGVLRGVDLSEAELG--------------KRGLGDTKDREPPFRECG-TMNGAQI
MSTRG.40154.1    GKSHTMGILRGLDIPAVDHQDPSLLQEAWRSTAEKERRGTSRNLESTVRGYRKPCTSVRP
                 #############                     ###############           


                        250       260       270       280       290       300
                 =========+=========+=========+=========+=========+=========+
Ec-10_005660     SSHNQTAPGIIPRALSRVFEHVQGSSVSSDRGSVADVSVKVKVSLLQIYNETVQDLLTPT
MSTRG.40154.1    YSHNQVDPGVIPRALSRVFDHES---RS------TDASAKVTVSLLQIYNETVQDLLSPM
                  ####################              #########################


                        310       320       330       340       350       360
                 =========+=========+=========+=========+=========+=========+
Ec-10_005660     APFGGESNGLIIRENPSLGFYVEGLREYSVRSHEEVCPGVERMVLSHAHGLRFDGPASIC
MSTRG.40154.1    SPFGGESIGLNIREDPSVGFFVEGLREHVVRSHQ--------------------------
                 #################################                           


                        370       380       390       400       410       420
                 =========+=========+=========+=========+=========+=========+
Ec-10_005660     ELPYIPHLHLLSPTFHFSTEAAELINLGLQQRTTSRTAKNDISSRSHTVLTVSLDQHGGI
MSTRG.40154.1    -------------------EAAALINSGLQQRTTARTSKNEISSRSHTVLTITLEHGSGL
                                    #########################################


                        430       440       450       460       470       480
                 =========+=========+=========+=========+=========+=========+
Ec-10_005660     GLFSTDPETDVANAARQHAGSRGKSKLMLVDLAGSERGRTSSIYAGEEPEADRLREAGYI
MSTRG.40154.1    ALFPSEPRAEATATAKLHTGDRGGSKLVLVDLAGSERGTRPKAYAQEESEPDRLREAGYI
                 ############################################################


                        490       500       510       520       530       540
                 =========+=========+=========+=========+=========+=========+
Ec-10_005660     NQSLSALGNVVAALGQAELDRPHIPFRDSKLTRILADSLGGDANAALVATVGPAPQNQAE
MSTRG.40154.1    NQSLSALGNVVAALGKPKADRPHIPFRDSKLTRILADTLGGNTNAALIATIGPAPQDQAE
                 ############################################################


                        550       560       570       580       590       600
                 =========+=========+=========+=========+=========+=========+
Ec-10_005660     TLSTLVFASRCMRVQSNPVRHEEHEEMEATARLQAQLSSMQREFCKREALQRSRYEMVVT
MSTRG.40154.1    SLSTLVFASRCMRVRNRPAKHEEGVEKETTLRLQAQLSSMQREFCRREALQQSRYETVVT
                 ############################################################


                        610       620       630       640       650       660
                 =========+=========+=========+=========+=========+=========+
Ec-10_005660     RLAQELEHARKQAASGRTAHRLPDQEAPAAAAPLMKNLKSSEEVHAREEAPGGTTSRGSA
MSTRG.40154.1    RLAQELEYARNKMTSAGASGWSADLKHRAPAVNFTTGGNPPKCSKCRAASLCDEESTTSE
                 ###############################               ############# 


                        670       680       690       700       710       720
                 =========+=========+=========+=========+=========+=========+
Ec-10_005660     FVSDARENGTWNGSKCSPAVATGSSGDGQTDEAESNEGDEGRAGAFCPEKAKASYSAESI
MSTRG.40154.1    TPQYHSFTATPAAARSDVGRQTDGTGSTDGGWCPPVLYRGDGSDTAVGEMTASVGTDTTI
                                                                            #


                        730       740       750       760       770       780
                 =========+=========+=========+=========+=========+=========+
Ec-10_005660     PESRGGASAIEADSTAWEMVGAALRTLEVVGQKGAQVLRRARHASDATPVDRRGR-----
MSTRG.40154.1    EGEEGVTVAVGHESVAWEMLGAAVRTLEVVRHKGAEVVRQAREAREAGDAARQETRLGQS
                 ####################################################        


                        790       800       810       820       830       840
                 =========+=========+=========+=========+=========+=========+
Ec-10_005660     --QARVDESSTLEEVASETIEHIPRNKVQILQRLTQSRKLCEEQWVEAGEALSSALRETT
MSTRG.40154.1    ASSAPLDEAGSVAEATPRTDEQISCMKIQILQRLGHLRELCERQWSEAGAILSSAVRDKR
                    #########################################################


                        850       860       870       880       890       900
                 =========+=========+=========+=========+=========+=========+
Ec-10_005660     RPGLL--------------------------------------------PARRK------
MSTRG.40154.1    RMQEMLRTEARLRHREETKARRLSGALRYLLDDGECARGDDLTPIPHEMPEGRQGSETEL
                 #                                                           


                        910       920       930
                 =========+=========+=========+
Ec-10_005660     ------------MKSQAITMRLNKIFVFTP
MSTRG.40154.1    ASPRFPNDINAGDQEQGCGLRADEKF----
                                ###########
```

```
Parameters used
Minimum Number Of Sequences For A Conserved Position: 2
Minimum Number Of Sequences For A Flanking Position: 2
Maximum Number Of Contiguous Nonconserved Positions: 8
Minimum Length Of A Block: 10
Allowed Gap Positions: None
Use Similarity Matrices: Yes
```

```
Flank positions of the 9 selected block(s)
Flanks: [149  193]  [215  229]  [242  261]  [276  333]  [380  631]  [647  659]  [720  772]  [784  841]  [916  926]  

New number of positions in Ec-10_005660.aa.fasta.trans.aa_ali.fasta-gb:  525  (56% of the original 930 positions)
```
